# Progress in Neurology

**Published:** 1904-03-12

**Authors:** 


### Progress in Neurology.

**Tumours of the Brain.**—Fraenkel and Hunt[1] record five cases of tumour of the ponto-cerebellar space. These are neuro-fibromata growing from the acoustic, or more rarely the trigeminal, nerve. They vary in size from a cherry to a hen's egg, are of fibrous consistency and distinctly encapsuled. A small remnant of the nerve-trunk, a few blood-vessels, and loose meningeal adhesions form the only connections of the tumour with its surroundings, so that it may be lifted with ease from its attachments, leaving a corresponding excavation in the structures composing the ponto-medullo-cerebellar space. Histologically, the tumours are neuro fibromata; in the more advanced stages the growth assumes a sarcomatous nature. The essential feature distinguishing these tumours from those originating within the substance of the brain stem or cerebellum is the early appearance of symptoms referable to a single cranial nerve; these may precede, for a considerable time, any other focal or general symptom. Hence tinnitis aurium with progressive diminution of hearing, attacks of Ménière's syndrome, obstinate attacks of facial neuralgia, may prove points of diagnostic importance. The conductivity of the nerve fibres is not always completely abolished, owing to the histological peculiarities of the growth. With the increase in size of the tumour, the neighbouring parts show evidences of pressure. Ataxia, tendency to deviate or fall to the same or opposite side, nystagmus, irregularities in size and reaction of pupils, paralysis of associated movements of the eyeballs, crossed paralysis of the extremities, facial paralysis of the peripheral type may occur. The diagnosis of the condition rests on the symptom-complex of tumour of the posterior fossa of the skull, preceded by well-marked and long-continued symptoms referable to disease of the fifth or eighth nerves. Removal of the growth by operative procedure offers the only hope. Because of the loose attachment of these tumours to their surroundings we may hope that, in spite of the unfavourable location, a perfected surgical technique will improve the prognosis.

**Relief of Pain in Tumours of the Brain.**—Browning[2] advocates the use of vascular depressants for the relief of pain in inoperable tumours of the brain. Some cases can be relieved by trephining, even though the tumour cannot be removed. A small number respond somewhat to thyroid extract, and many are temporarily benefited to an appreciable extent by iodide. When these means are exhausted, the cases usually end by making continuous use of opiates in some form; but this has objections, consciousness isdulled, and the resulting constipation in the long run aggravates the pain. In such cases the employment of opiates can be often staved off by the administration of such depressants as aconitia, veratrum or gelsemium, in doses sufficient to soften the pulse. The dosage in any particular case is kept where it does not dangerously reduce the pulse on the one hand and yet controls the pain on the other. The plan does not completely stop the pain, but it often holds it within bearable limits, even for many months, accomplishing as much as symptomatic trephining and that without any of the operative drawbacks.

**Argyll-Robertson Pupil.**—The relations of the presence of the Argyll-Robertson pupil to past syphilitic infection are discussed by Clarke[3] in an interesting paper. It is usually stated that this occurs in tabes dorsalis, general paralysis, and acquired or congenital syphilis, and that it is only exceptionally found in other diseases of the nervous system. From an experience of 37 cases Clarke comes to the conclusion that a previous syphilitic affection is not sufficient without some further change to lead to the Argyll-Robertson phenomenon. Where the mischief is confined to the ordinary visceral lesions of that disease—gummata, gummatous meningitis and syphilitic endarteritis—the sign is not typically present. In similar cases where it is present and in cases with a history of past syphilis where it occurs as an isolated sign, it is evidence of a further degenerative process at work within the nervous system. This degenerative process is to be regarded in the large majority of cases as a parasyphilitic one, though in a certain proportion proof of this is wanting, and there is possibly some other cause at present obscure. This degeneration may remain stationary at an early stage without further development for long periods. The Argyll-Robertson phenomenon is thus an even more important sign of disease than it has been previously considered.

Headaches.—Auerbach[4] says that headaches are to be known as "functional" only after exclusion of organic disease. First one excludes brain tumour, then arterio-sclerosis, syphilis, refractive and other ocular headaches, nasal and oral disease and dental caries, nephritis, anæmia, dyspepsia and constipation. Of residual "functional" headaches we know (1) neuralgic, (2) neurasthenic, (3) hysterical, (4) migraine, and (5) nodule, headaches. In supra-orbital neuralgia the pain is never in but always on the head, unilateral and accompanied by tenderness in pressure on the affected nerve. In neurasthenia the headache is not a pain but a pressure sensation often in the form of a band. Hysterical headaches occur principally in morbidly suggestible women and are generally associated with complaints of other bodily sensations. In migraine the diagnosis is generally easy, the attacks are often hereditary and begin in childhood, and they are definitely periodical. Nodule headaches occur mostly in women of middle or advanced age, who were free from headache in youth. The pain is persistent and violent, and generally originates in the occipital and cervical regions, not ceasing at night, and rarely associated with nausea. It is always bilateral. "Catching cold" bears a definite and decided relation to the disease. Examination reveals millet-seed to bean-sized nodules in the subcutaneous tissue of the occiput and head, which are tender on pressure. Bromides do no good—unlike their action in migraine—whilst massage cures. The duration of treatment varies from three or four weeks to a couple of months. If the condition is associated with anæmia or neurasthenia, tonics such as quinine, arsenic and iron should also be administered.

[1] Med. Rec., Dec. 26, 1903. [2] Jour. of Nerv. and Mental Dis., Nov. 1903. [3] Brit. Med. Jour., Dec. 26, 1903. [4] Med. Rec., Nov. 21, 1903.

